# Oxidative stress, ceruloplasmin and neopterin biomarkers in dromedary camels with clinical endometritis

**DOI:** 10.1590/1984-3143-AR2022-0035

**Published:** 2022-09-12

**Authors:** Wael El-Deeb, Mohammed Ali Abdelghani, Abdulrahman Alhaider, Mahmoud Fayez

**Affiliations:** 1 Al Bilad Bank Scholarly Chair for Food Security in Saudi Arabia, Deanship of Scientific Research, Vice Presidency for Graduate Studies and Scientific Research, Al Ahsa, Saudi Arabia; 2 Department of Clinical Sciences, College of Veterinary Medicine, King Faisal University, Al-Ahsa, Saudi Arabia; 3 Department of Internal Medicine, Infectious Diseases and Fish Diseases, Faculty of Veterinary Medicine, Mansoura University, Mansoura, Egypt; 4 Department of Theriogenology, Faculty of Veterinary Medicine, Assuit University, Assuit, Egypt; 5 Al Ahsa Veterinary Diagnostic Laboratory, Ministry of Environment, Water and Agriculture, Al Ahsa, Saudi Arabia

**Keywords:** camel, ceftiofur, endometritis, neopterin, ceruloplasmin

## Abstract

The purpose of this study is to investigate the role of some oxidative stress (OS), ceruloplasmin (Cp), and neopterin (NPT) as diagnostic biomarkers for dromedary camels endometritis as well as to explore the impact of ceftiofur treatment on endometritis. Camels were categorized into two groups; healthy control group (n = 20) and endometritis group (n = 60). She-camels with clinical signs of endometritis (CE) received 6.6 mg/kg BW of ceftiofur (i/m). On days 7, and 14, she-camels were evaluated and clinical cure or failure to cure was determined. The comparison of the groups for OS demonstrated that endometritis caused an increase in serum malondialdehyde (sMDA), Cp, and NPT levels (P<0.05), but decreased serum levels of superoxide dismutase (SOD), catalase (CAT), and glutathione (GSH) (P<0.05). The most prevalent pathogens involved in the etiology of CE are *Arcanobacterium pyogenes, Streptococcus pyogenes, and Staphylococcus aureus.* All examined biomarkers demonstrated a high degree of recognition between CE camel and healthy controls (the area under the curve (AUC) was 95.9 for NPT). A higher proportion of camels with CE that were treated with ceftiofur (90%, P<0.0001) showed clinical cure by the first dose, while 10% required a second dose. In conclusion, CE causes increased oxidative reactions and decreased antioxidant defense competence. Subsequently, the alteration in that balance that was represented by the biomarkers of OS could be beneficial for clinical practice and basic clinical research. Additionally, all trials demonstrated the efficacy of ceftiofur for the treatment of CE in she-camel.

## Introduction

The reproductive disorders of the dromedary camel have not been studied as thoroughly as they are in the bovine. The prevalence of pathological disorders among reproductive organs had differences, where the highest proportion was observed in the uterus (Belina et al., 2021). Endometritis is one of the most common disorders, causing decreased fertility and high economic losses (Wernerry and Kumar, 1994). A bacterial infection induces an inflammatory response as well as an increase in oxidative stress (OS). OS is defined as an imbalance between increased production of oxidant species and/or decreased efficacy of the antioxidant defense system (Gosmaro et al., 2013) leading to macromolecule damage, lipid peroxidation, protein cross-linking, DNA damage, and changes in the growth and function of cells (Ehsaei et al., 2015). OS is characterized by elevated levels of reactive oxygen species (ROS) and is currently diagnosed to be a noticeable highlight of numerous acute and chronic diseases (Gosmaro et al., 2013; El-Deeb and Buczinski, 2015; El-Deeb and Elmoslemany, 2015).

However, conclusive proof for this affiliation has frequently been authorized with biomarkers to be used to evaluate OS status. The emphasis is now being placed on biomarkers of OS, which might be objectively measured and evaluated as signs of biological phenomena, pathogenic processes, or pharmacologic reactions to restorative mediation (Andrei et al., 2016). To be a predictor of disease, a biomarker must be approved, where; the approval criteria incorporate inborn qualities such as specificity, and sensitivity (Dalle-Donne et al., 2006). The foremost instinctive objectives for a biomarker are to analyze symptomatic and pre-symptomatic infection, and the effectiveness of the perfect biomarker lies in its capacity to supply early signs of infection and/or its development (Andrei et al., 2016).

Moreover, there are multiple options for the treatment of infections endometritis. Ceftiofur, a third-generation cephalosporin, is utilized as an antimicrobial agent in veterinary medicine that the only antibiotic approved by the Food and Drug Administration (FDA) for treatment of metritis that does not demand milk withdrawal, as well as it, enhanced milk production and fertility (Jeon et al., 2021). Ceftiofur is known to have broad-spectrum activity, and its efficiency is high against approximately all gram-positive and gram-negative bacteria (Hornish and Katarski, 2002). The efficacy of ceftiofur for the treatment of metritis has been previously described and the cure rate of ceftiofur for metritis in cows is estimated by clinical resolution and has been reported to range from 67 to 85% (Chenault et al., 2004; Oliveira et al., 2020).

To the best of the authors’ knowledge, there have been no studies conducted on the role of these biological markers in disease diagnosis and pathogenesis, and the effects of ceftiofur on endometritis of she-camel. In view of these considerations, the objectives of the present study are to investigate the relevance of determine diagnostic biomarkers of endometritis in camelids production and to determine the efficacy of ceftiofur in the treatment of CE.

## Methods

### Ethical approval

All experimental procedures used in the current study were approved by the guidelines of Ethics Committee at King Faisal University, Saudi Arabia (KFU/2019-10-01).

### Animals and endometritis diagnosis

A total of 80 she-camels were used in this study. She-camels were clinically investigated in the Veterinary Teaching Hospital (VTH), and Camel Research Center (CRC), King Faisal University, Saudi Arabia. The soundness of animals was evaluated. She-camels without systemic lesions or deformities were classified as suitable for the experiment, and those showing alterations or pathological lesions rather than endometritis were excluded. According to clinical examination, and the laboratory analysis, the camels were categorized into two groups. The she-camels of the first group were comprised of healthy individuals (n = 20; control group) however the other group were she-camels with clinical endometritis of different grades (n = 60; Endometritis group). The selection of the control group was based on full clinical examination of she-camels and special clinical examination of the uterus. The healthy she-camels were examined in the VTH and CRC for a routine examination before breeding season.

For each she-camel, the ovaries, uterus, and cervix were examined through rectal palpation and by trans-rectal ultrasound (7.5 MHz transducer, Aloka, Japan) with manual vaginal exploration. The camels were diagnosed with endometritis following criteria described by Sheldon et al. (2006). These criteria include repeat breeding, vaginal mucopurulent/purulent discharge, the uterus enlarged and thickened with intrauterine fluid (Catarrhal, mucopurulent, or purulent) accumulation that contains echogenic particles. The camels without endometritis were considered control.

The endometritis grade was classified as moderate endometritis (n = 56): catarrhal (turbid mucus) or mucopurulent (turbid mucus with flecks of pus) vaginal discharge, and severe endometritis (n = 4): purulent vaginal discharges. The vaginal discharge was graded using a 0 to 2 scale: 0 = normal uterine discharge; 1, moderate = turbid mucus or turbid mucus with flakes of purulent exudates in the uterine discharge; 2, sever = the uterine discharge is made up of purulent exudates [adapted from Sheldon et al. (2006)]. Clinical endometritis was defined as any she-camel presenting a score of 1 or 2 (mucopurulent or worse vaginal discharge) at the time of exam.

### Oxidative stress, ceruloplasmin (Cp) and neopterin (NPT) biomarkers determination

Serum samples were collected and stored at −20 °C until biochemical analyses were performed. Detection of serum malondialdehyde (sMDA), reduced glutathione (GSH), super oxide dismutase (SOD), and Catalase (CAT) levels was estimated by colorimetric method using available test kits (Bio-diagnostic, Egypt) as previously described (El-Deeb et al., 2019; El-Deeb et al., 2020; El-Deeb et al., 2021). The reaction of MDA with thiobarbituric acid (TBA), forms a TBA-reactive product when it is placed in acidic medium at 95 °C for 30 min. The TBA-reactive product is pink in color and possesses a measurable absorbance at 534 nm. Lipid peroxidation in serum was expressed as nmol of sMDA per g serum protein (Shimadzu AA-6800 atomic absorption spectrophotometer, Koyoto, Japan). The levels of GSH were measured at 405 nm achievable through reduction of 5,5-Dithiobis (2-nitrobenzoic acid) with GSH and measurement of the yellow product produced. The SOD assay depends on the enzyme’s ability to inhibit the reduction of nitroblue tetrazolium dye, which is mediated by phenazine methosulfate. The CAT levels were measured based on the reaction of the catalase enzyme with a specified amount of hydrogen peroxide, as described by Aebi (1984). Serum NPT concentrations were measured using ELISA kits (Bovine NPT ELISA Kit, Fine Test, Wuhan Fine Biotech, Wuhan, China) as instructed by the manufacturer. Based on the method described by Sunderman and Nomoto (1970), the ceruloplasmin activity was determined by measuring the phenylenediamine oxidase activity (Shimadzu AA-6800 atom absorption spectrophotometer; Shimadzu, Kyoto, Japan).

### Collection of uterine swab for bacteriological examination

A double-guarded uterine instrument was used to collect swabs from animals diagnosed with CE. Uterine swabs were collected using a previously validated method (Sheldon et al., 2002a; Williams et al., 2007). Briefly, the vulvas was thoroughly cleaned. Then, the double guarded instrument containing the swab was inserted through the vagina and cervical canal into the lumen of the uterus, guided by palpation per rectum. Within the uterine body, the cotton swab was extruded from the double guard tube and brought into contact with the endometrium by gentle pressure per rectum about 2-3 cm from the uterine bifurcation. The rotated for 15-30 seconds before being withdrawn carefully into the protected tube of double-guarded instrument, to avoid vaginal contamination, and removed form uterus.

### Bacteriological examination

Each swab was streaked on to MacConkey agar, sheep blood agar, Salmonella-Shigella agar (Oxoid, England) and incubated at 37 oC for 48 hours. Bacterial colonies were examined for their morphology, macroscopic characteristics, Gram staining, catalase and oxidase activities. Colonies were purified on brain heart infusion agar (Oxoid, England) and subjected to biochemical identification by the automated Vitek compact system using GP, GN and AN identification kits (bioMérieux, France).

### Ceftiofur treatment

The previous study in she-camel (Kandeel et al., 2021) reported that one dose of ceftiofur crystalline acid-free form (Ceftiofur-CAF; i/m) at a dose rate of 6.6 mg/kg attained serum level above 0.2 μg/ml for 7 days, and a single dose gives 7 days cure against highly susceptible bacteria. Consequently, treatment was carried out by using a dose 6.6/ mg/kg BW (i/m) of Ceftiofur-CAF 200 mg/ml suspension (Excede, Zoetis Inc, NJ, USA). The response to treatments was assessed on days 7 and 14 d post-therapy administration at the second examination for she-camel with CE. The clinical resolution was defined as the absence of mucopurulent or worse uterine discharge at the second examination i.e. animals with clear, translucent, or no mucus. Clinical cure or failure to cure was determined. The second dose of ceftiofur-CAF after 7 days for those animals did not respond to the first injection.

### Experimental design

**Figure 1:**
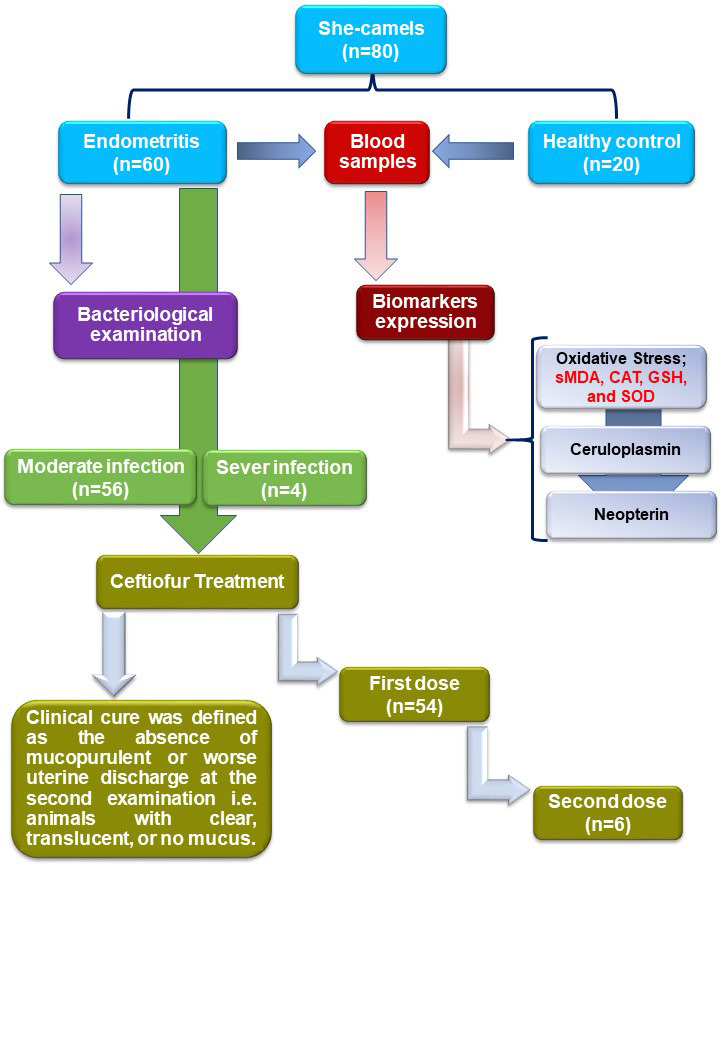
Flowchart showing study design and the number of she-camels with and without clinical endometritis.

The experimental design was shown in Figure 1. The flowchart showing study design, number of she-camels with and without clinical endometritis.

### Statistical analysis

All parameters were expressed as mean ± SD. Comparisons in mean were performed by Kruskal–Wallis ANOVA on Ranks followed by Dunn’s multiple comparisons. The different means were significant at P<0.05. Statistical analysis was performed using JMP software version 11.0.0 (SAS Institute, Cary, NC, USA). The response to the ceftiofur was analyzed by Chi-square test. The correlation between parameters was evaluated using Spearman’s rank correlation test. Each assay’s diagnostic accuracy was evaluated by creating the ROC (receiver operator characteristics) curve and determining the area under the curve (AUC). An AUC of 0.7 to 0.9 was considered moderately accurate, an AUC of >0.9 highly accurate, and an AUC of 1 perfect (Gardner and Greiner, 2006). Graphpad Prism v5 software (Graphpad Software, Inc., San Diego, CA) was used to draw the figures.

## Results

### Oxidative stress (OS), ceruloplasmin (Cp) and neopterin (NPT) biomarkers expression

**Figure 2:**
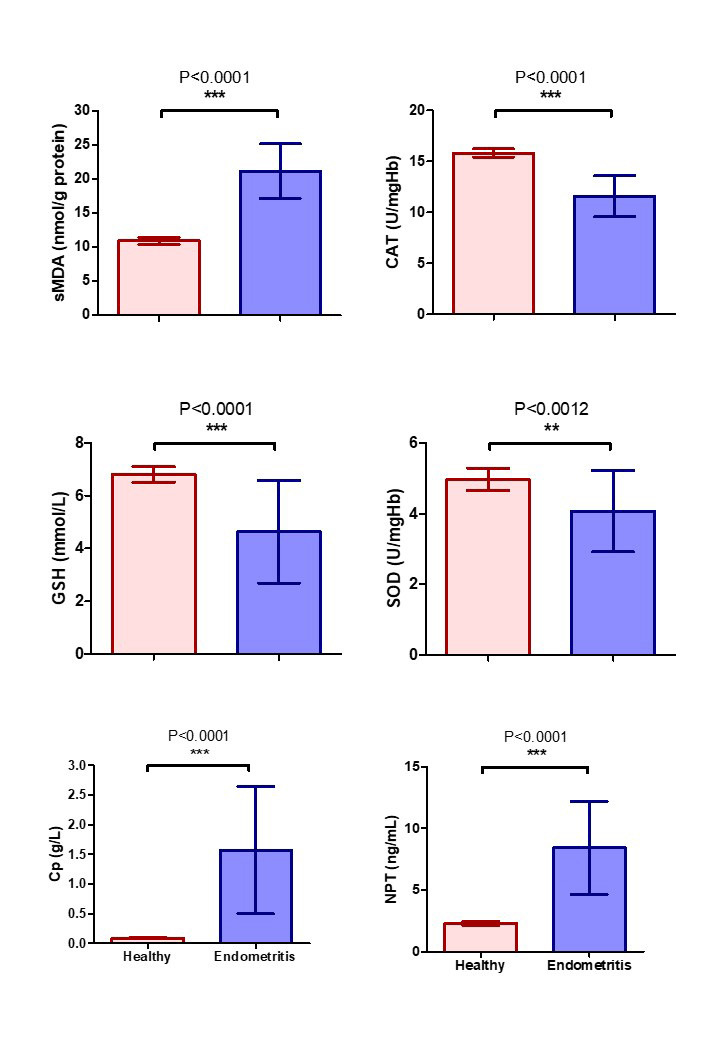
Oxidative stress, ceruloplasmin, and neopterin biomarkers expression in she-camel with and without clinical endometritis (Control). Serum malondialdehyde (sMDA), catalase (CAT), reduced glutathione (GSH), super oxide dismutase (SOD), ceruloplasmin (Cp), and neopterin (NPT). Data represented in mean ± SD. Differences between groups were calculated using the t-test. *Asterisk indicates a significant difference between the experimental groups (P < 0.05).

The results of OS markers, Cp, and NPT expression in she-camel with and without clinical endometritis (CE) were set out in Figure 2. The mean sMDA and Cp values in she-camel with endometritis were consistently higher than that of control (P<0.05). In contrary, the mean values of CAT, GSH, and SOD were lower in the endometritis group (P<0.05) compared with the healthy control. The values of NPT were impressively higher (P<0.05) in she-camel with CE than in healthy control.

The correlation coefficients (*r*) between biomarkers were analyzed using Spearman's correlation coefficient matrix in she-camel with CE and control ones (Table 1). The highest negative correlation was observed between CAT and Cp (*r* = - 0.64), while the highest positive correlation was observed between CAT and GSH (*r* = 0.56).

**Table 1:** Correlation matrix between oxidative stress, ceruloplasmin, and neopterin biomarkers in healthy and she-camels with clinical endometritis.

	**sMDA**	**CAT**	**GSH**	**SOD**	**Cp**	**NPT**
sMDA	1					
CAT	-0.41	1				
GSH	-0.54	0.56	1			
SOD	-0.55	0.45	0.52	1		
Cp	0.49	-0.64	-0.55	-0.5	1	
NPT	0.52	-0.58	-0.41	-0.58	0.45	1

sMDA: serum malondialdehyde; CAT: catalase; GSH: reduced glutathione; SOD: super oxide dismutase; Cp: ceruloplasmin; NPT: neopterin.

**Figure 3:**
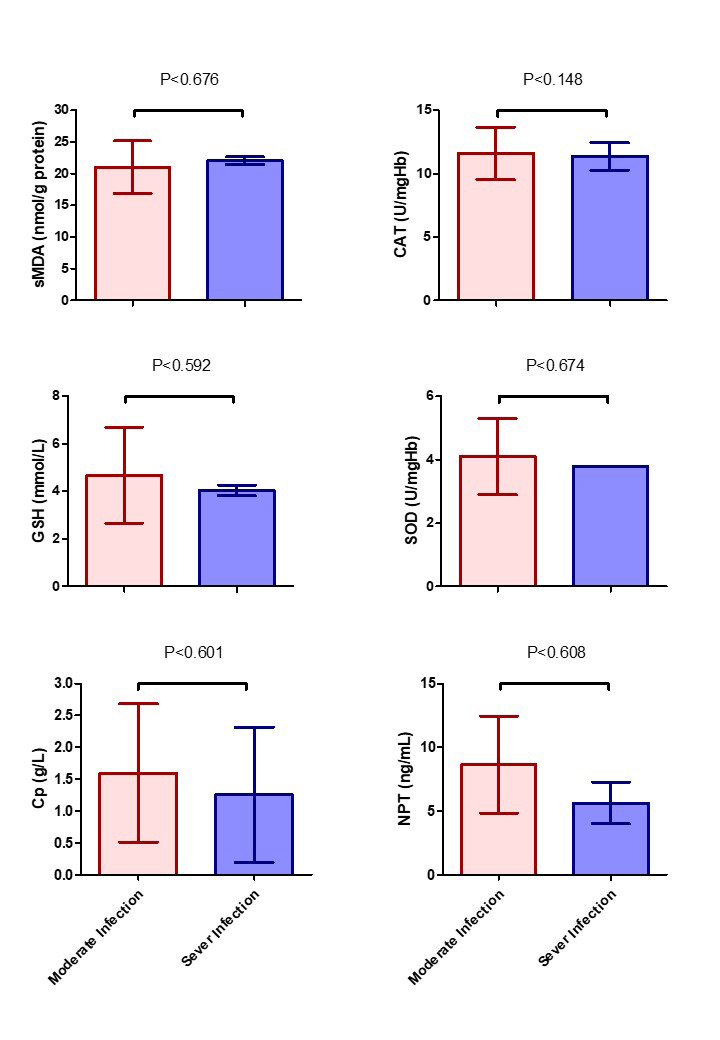
Oxidative stress, ceruloplasmin, and neopterin biomarkers expression in she-camel with and moderate and sever endometritis. Serum malondialdehyde (sMDA), catalase (CAT), reduced glutathione (GSH), super oxide dismutase (SOD), ceruloplasmin (Cp), and neopterin (NPT).

The examined biomarkers did not differentiate the peculiar bacterial infections, where, no differences (P > 0.05) were noted between endometritis pathogen-species and changes in biomarkers expression. Additionally, no differences (*P > 0.05*) were observed in biomarkers expression between moderate and sever endometritis (Figure 3).

**Figure 4:**
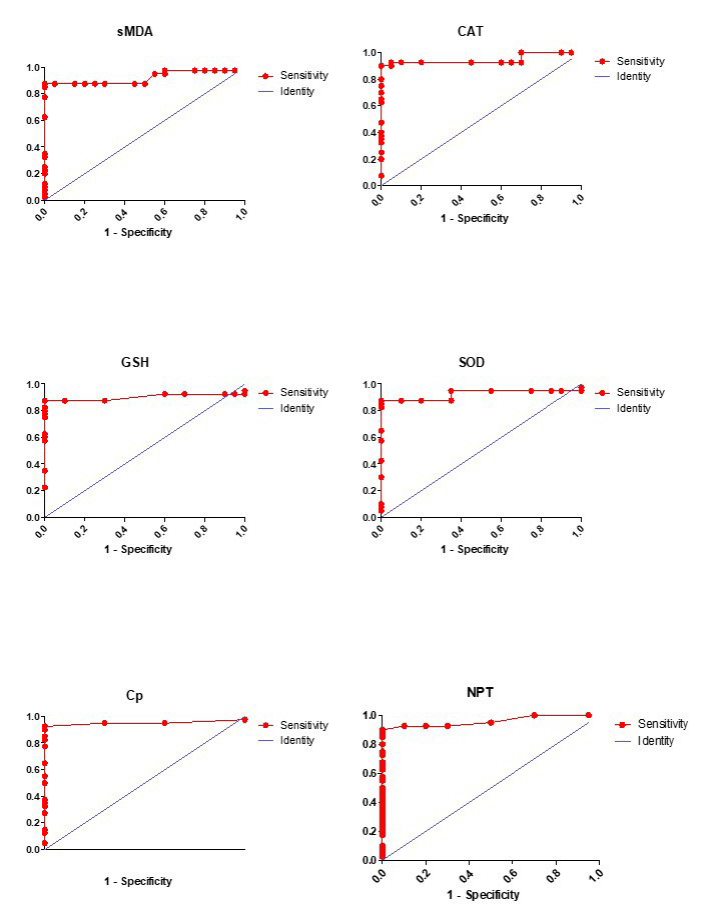
Receiver operating characteristic (ROC) curve analysis of oxidative stress, ceruloplasmin, and neopterin biomarkers in she-camel with clinical endometritis. Serum malondialdehyde (sMDA), catalase (CAT), reduced glutathione (GSH), super oxide dismutase (SOD), ceruloplasmin (Cp), and neopterin (NPT).

The ROC curves were made and AUC was determined in order to evaluate the parameter’s accuracy in arrange to distinguish between she-camel with and without endometritis (Figure4). The optimal cut-off value of NPT, Cp, and CAT when using ROC analysis to distinguish between she-camel with and without endometritis were 2.55 ng/mL, 0.11 g/L, and 14.40 (U/mgHb), respectively. The other parameters were very similar to each other and showed accurate diagnostic performance (sensitivity > 0.87) (Table 2).

**Table 2:** Diagnostic test characteristics of oxidative stress, ceruloplasmin, and neopterin parameters in healthy and she-camel with clinical endometritis.

**Parameters**	**Threshold**	**Diagnostic characteristics (%)**			**Accuracy**	**AUC**
		**Se (95% CI)**		**Sp (95% CI)**		
sMDA (nmol/g protein)	≥ 15.98	87.5 (73.2% to 95.8%)		100 (83.2% to 100%)	91	0.921
CAT (U/ mgHb)	≤ 14.40	90.0 (76.3% to 97.2%)		100 (83.2 to 100%)	93	0.946
GS H (mmol/L)	≤ 5.60	85.0 (73.2% to 95.8%)		70 (45.7% to 88.1%)	86	0.902
SOD (U/mgHb)	≤ 4.35	87.5 (73.2% to 95.8%)		90 (68.3% to 98.7%)	88	0.923
Cp (g/L)	≥ 0.11	95.0 (83.1% to 99.4%)		100 (83.2 to 100%)	91	0.951
NPT (ng/mL)	≥ 2.55	90.0 (76.3% to 97.2%)	100 (83.2 to 100%)		93	0.959

sMDA: serum malondialdehyde; CAT: catalase; GSH: reduced glutathione; SOD: super oxide dismutase; Cp: ceruloplasmin; NPT: neopterin.

### Uterine bacterial isolates

The bacteria colonizing the uterus in camels are shown in Table 3. The most prominent bacterial isolates from the uterus were made up of 11 (18.3%) *Arcanobacterium pyogenes*, 10 (16.6%) *Streptococcus pyogenes*, 8 (13.3%) *Staphylococcus aureus*, *Escherichia coli*, and *Salmonella species*. Out of these numbers, 4 (6.6%) were *Corynebacterium pyogenes* and *Klebsiella species*, 5 (8.3%) *Arcanobacterium pyogenes* + *Proteus mellitus*, and 2 (3.3%) *Escherichia coli* + *Proteus mellitus*.

**Table 3:** Uterine bacterial isolates from the she-camel with clinical endometritis.

**Isolates**	**She camels (n = 60)**	
	**n**	**%**
*Streptococcus pyogenes*	10	16.6
*Staphylococcus aureus*	8	13.3
*Arcanobacterium pyogenes*	11	18.3
*Corynebacterium pyogenes*	4	6.6
*Escherichia coli*	8	13.3
*Salmonella species*	8	13.3
*Klebsiella species*	4	6.6
*Arcanobacterium pyogenes + Proteus mellitus*	5	8.3
*Escherichia coli + Proteus mellitus*	2	3.3

### Effect of ceftiofur antibiotic

The rates of resolution of clinical signs were high for both initial and second treatments. A higher proportion of she-camel with CE that were treated with ceftiofur (90%, P<0.0001) scored zero (clear mucus; clinical cure) after the first dose, while 10% required a second dose of ceftiofur. 88% of moderate infections are fully cured by the first dose of ceftiofur compared to severe infections (2%).

Additionally, no significant differences (P > 0.05) were observed in the proportion of she-camel that responded to the second dose, with moderate (5%) or severe infection (5%).

## Discussion

A number of diseases are associated with OS, wherein the production of ROS plays a critical role in bacteria killing (Su et al., 2019). Among the many aldehydes that can be formed as secondary products during lipid peroxidation, MDA appears to be the most mutagenic product of lipid peroxidation, and the higher blood levels of free radicals cause higher MDA production (Gaschler and Stockwell, 2017). Measurement of the MDA gives an appropriate marker of lipid peroxidation (Nielsen et al., 1997), and consistently, the results of this study showed that sMDA significantly increased in she-camel with CE, indicating high level of lipid peroxidation due to inflammatory process.

The SOD converts superoxide radicals into hydrogen peroxide and oxygen, while the CAT and GSH degrade hydrogen peroxide into water and oxygen (He et al., 2017; Wang et al., 2018). The present study showed a significant decrease in the activity of SOD, CAT, and GSH in she-camel with CE as compared to healthy control. The decline in these antioxidant enzyme concentrations revealed that they were conscientiously involved in neutralizing and detoxifying free radicals that are produced during OS. The reduced antioxidant enzyme levels are thought to be for their implementation in the conversion of harmful free radicals to harmless molecules, such as the conversion of hydrogen peroxide that was generated throughout the SOD reaction to water. These results are consistent with previously reported data by Hanafi et al. (2008) who reported that the blood of buffalo-cows having CE showed increased MDA and decreased CAT and GSH. Moreover, the rats with endometritis revealed an increase in MDA concentration and a decrease in the activity of CAT and GSH as compared to healthy control (Tiwari et al., 2017).

Macrophages and monocytes generate ROS and NPT when they are stimulated by IFN, which is considered a marker for macrophage activation. Hence, increased NPT levels are often detected in OS-related diseases, and it is reported that NPT may be an indirect indicator of OS induced by the immune system (Murr et al., 2002). Moreover, NPT is associated with the severity of disease and is considered a potential biomarker predicting adverse outcomes in numerous diseases (Peng et al., 2020). In our study, a significant elevation of serum levels of NPT has been shown in she-camels with CE. To our knowledge, this is the first study to demonstrate that serum NPT is a diagnostic biomarker for CE in she-camel.

Cp, produced mainly in the liver, is a positive-phase protein, which means its level alters in inflammation, infection, and trauma, which is mainly attributed to its antioxidant properties (Adamczyk-Sowa et al., 2016). Taken together, the findings mentioned above, indicate that it should be expected that Cp levels would increase with disease activity, in agreement with our results that showed a higher level of Cp was observed in she-camel with CE.

ROC analysis is a valuable method to evaluate diagnostic tests and is used to assess accuracy quantitatively. Herein, ROC analysis was used to estimate the ability of OS and NPT markers to differentiate between she-camel with CE and healthy controls. The OS and NPT biomarkers showed a high level of significance between endometritis and healthy control, which is consistent with the guidelines of diagnostic accuracy (Mandrekar, 2010). NPT showed the highest accuracy. Hence, it could be considered as an additional tool for diagnosing endometritis. NPT is thought to be biochemically dormant due to its half-life in the body (El-Deeb et al., 2020; El-Deeb et al., 2021). Therefore, diagnosis of NPT has a number of advantages over OS biomarkers that have a relatively short half-life and faster degradation, which is in agreement with the data reported by El-Deeb et al. (2021).

Ceftiofur is a β-lactam antibiotic that combines with the bacterial enzyme dd-transpeptidase, blocking the formation of crosslinks between this enzyme and peptidoglycan, which is pivotal for the formation of a rigid cell wall synthesis during bacterial binary fission. Disruptions in cell wall synthesis result in cell lysis and the death of the bacteria (Chambers, 2007). Ceftiofur has great potency against gram-positive and gram-negative bacteria and is resistant to β-lactamase, therefore blocking the action of these enzymes on the degradation of the β-lactam ring, which deactivates many antibiotics of this class (Hornish and Katarski, 2002). Most bacteria associated with metritis are susceptible to ceftiofur (Malinowski et al., 2011).

The most prominent pathogens involved in the etiology of endometritis in cows are *Arcanobacterium pyogenes* and *Escherichia coli* (Sheldon et al., 2002b) should be vulnerable to ceftiofur. Pathogens predominantly associated with endometritis in she-camel, i.e. *Arcanobacterium pyogenes*; suggested treatment protocols for clinical endometritis include the ceftiofur. Additionally, Galvão et al. (2009) reported that intrauterine infusion of ceftiofur positively affected uterine health in dairy cows. Erskine et al. (2002) evaluated the efficacy of intramuscular administration of ceftiofur to reduce the incidence of case-related death following severe clinical mastitis in lactating dairy cattle. He reported that intramuscular injection of ceftiofur for severe clinical mastitis cases reduced the proportion of cases that resulted in cow death or culling. The systemic administration of ceftiofur may be gained by the amelioration of deleterious effects of bacteremic-related pathogenesis, which has been reported to be occurred (Erskine et al., 2002). Haimerl and Heuwieser (2014) reviewed the literature conducted on the antibiotic therapy of puerperal metritis in dairy cows. They documented that more than 73.9% of the clinical trials used ceftiofur for the treatment of metritis, pneumonia, and lameness in cattle. The systemic injection of ceftiofur results in concentrations that exceed the minimum inhibitory concentrations for pathogens associated with uterine disorders (Drillich et al., 2006), and has an efficacious therapy for the treatment of acute postpartum metritis in dairy cows (Chenault et al., 2004). Lima et al. (2014) reported that the systemic ceftiofur administration was an efficient therapy for metritis in dairy cows and the subsequent effects on pregnancy at first insemination (P/AI). Furthermore, because the inflammatory process involves deeper layers of the uterus in CE and other genital tissues, systemic therapy would be necessary. The treatment of CE with ceftiofur antibiotics in she-camel has not been published in recent literature. The current study shows the effectiveness of ceftiofur in eliminating uterine pathogens in she-camel with CE. Furthermore, 90% of treated cases responded to a single dose of ceftiofur, while 10% from the final administration of a first dose treatment did not ensure resolution and required a second dose.

## Conclusion

It is well known that a reverse correlation exists between uterine infections and reproductive performance, thus, a healthy uterus can help to improve the fertility rate. The present study showed that the clinical endometritis she-camels have remarkable changes in serum OS, CP, and NPT levels, whereas, we observed higher levels of these biomarkers in she-camel with CE than healthy controls. Moreover, there was no significant difference between the levels of examined biomarkers between bacterial isolates infected endometritis she-camel. Clinical cure was remarkably high with ceftiofur administration, and ceftiofur was effective for the treatment of CE in she-camel. This knowledge may be used to diagnose disease earlier or to prevent it before it starts. These biomarkers can be used to investigate the effectiveness of the predominating medicines and to enhance the new medicines in dromedary camels.
